# Healthy lifestyle factors outweigh influence of APOE genetic risk on extending cognitively healthy life expectancy among Chinese older adults: evidence from a nationwide cohort study

**DOI:** 10.1002/alz.70090

**Published:** 2025-04-14

**Authors:** Longbing Ren, Fan Hu, Sebastian Walsh, Xurui Jin, Yang Hu, Shaojie Li, Yuling Jiang, Mingzhi Yu, Yifei Wu, Grace Yuange Zang, Keyang Liu, Huashuai Chen, Jing Sun, Yan Zhang, Kokoro Shirai, Yi Zeng, Quincy M. Samus, Gill Livingston, Yao Yao

**Affiliations:** ^1^ School of Public Health Peking University Health Science Center Beijing China; ^2^ Center for Healthy Aging Transdisciplinary Sciences, China Center for Health Development Studies Peking University Beijing China; ^3^ Division of Psychiatry University College London London UK; ^4^ Department of Endocrinology, The Second Medical Center & National Clinical Research Center for Geriatric Disease Chinese PLA General Hospital Beijing China; ^5^ Cambridge Public Health University of Cambridge Cambridge UK; ^6^ MindRank AI Ltd. Hangzhou Zhejiang China; ^7^ Department of Health Policy and Management, Bloomberg School of Public Health Johns Hopkins University Baltimore Maryland USA; ^8^ Department of Public Health, Graduate School of Medicine Osaka University Suita Osaka Japan; ^9^ School of management Xiangtan University Xiangtan Hunan China; ^10^ School of Nursing Peking University Beijing China; ^11^ School of Health Service Management Anhui Medical University Hefei Anhui China; ^12^ Center for the Study of Aging and Human Development and Geriatrics Division Medical School of Duke University Durham North Carolina USA; ^13^ Department of Psychiatry and Behavioral Sciences School of Nursing, Johns Hopkins School of Medicine Baltimore Maryland USA; ^14^ State Key Laboratory of Vascular Homeostasis and Remodeling Peking University Beijing China; ^15^ Key Laboratory of Epidemiology of Major Diseases (Peking University) Ministry of Education Beijing China

**Keywords:** APOE genotype, cognitive impairment, healthy lifestyles, life expectancy, low‐ and middle‐income countries

## Abstract

**INTRODUCTION:**

Understanding the interplay between genetic factors and lifestyle choices in cognitive health is crucial for enhancing late‐life quality. This study examines the effects of Apolipoprotein E (APOE) genotypes and healthy lifestyles on life expectancy with and without cognitive impairment (CI) in Chinese older adults.

**METHODS:**

Data from 6488 participants aged at least 65 in the Chinese Longitudinal Healthy Longevity Survey (CLHLS) were analyzed using continuous‐time three‐state Markov models. Cognitive function was assessed with the Mini‐Mental State Examination (MMSE).

**RESULTS:**

APOE ε4 allele carriers had a higher risk of transitioning from cognitively healthy (CH) to impaired, while ε2 carriers had a reduced risk of transitioning from healthy to death. Participants with 4 or 5 healthy lifestyle factors experienced significant protective effects, extending the cognitively healthy life expectancy.

**DISCUSSION:**

These findings underscore the importance of promoting healthy lifestyles to delay cognitive decline, regardless of genetic predispositions, particularly in the Asian context.

**Highlights:**

- Compared with ε3 homozygotes, APOE ε4 carriers in China have a higher risk of transitioning from CH to CI, and APOE ε2 carriers with CH have a lower risk of transitioning to death.
- Healthy lifestyles can extend life expectancy, primarily extending CH life expectancy.
- Healthy lifestyles reduce the risk of CI and delay its onset in later life, regardless of APOE genetic risk.

## 1 BACKGROUND

China has the largest population of individuals with dementia worldwide, and this number is expected to continue increasing.^1^ Apolipoprotein E (APOE) is the most common genetic risk factor for age‐related cognitive impairment (CI) and dementia.^2^, ^3^ Carriers of the APOE ε4 allele exhibit markedly higher rates of early amyloid beta (Aβ) accumulation, especially those homogeneous for the ε4/ε4 genotype.^4^ Conversely, the ε2 allele is associated with lower Aβ accumulation.^5^, ^6^ APOE genotypes and cognitive function are influenced by sex and ethnicity.^7^, ^8^ Although the ε4 allele is more common among Black individuals, its association with AD risk is stronger among non‐Hispanic Whites.^3^, ^9^ Recent research on the ε2 allele around sex‐specific effects has inconsistent findings, with different ethnic groups with a protective effect reported in non‐Hispanic White males,^10^ a female‐specific protective effect among Whites, and a male‐specific one among Blacks.^8^ However, Asians, despite having the largest older populations,^11^ have not been included in these studies. To date, no large‐scale longitudinal studies have investigated the relationship between APOE genotypes (particularly APOE ε2) and cognitive function in East Asian older generations.

Recent reports and studies have indicated that maintaining a healthy lifestyle can slow cognitive decline and reduce nearly half of the risk of dementia in late life.^12^, ^13^, ^14^, ^15^, ^16^ Healthy lifestyles can also extend life expectancy.^17^, ^18^, ^19^ In a study from the United States, healthy lifestyles were shown to extend life expectancy and delay the onset of dementia, indicating that healthy lifestyles may compress the duration of life lived with dementia.^20^ However, the benefits of a healthy lifestyle for cognitive health, regardless of APOE ε4 carrier status, remain inconclusive.^12^, ^21^, ^22^, ^23^, ^24^ Moreover, these studies often exclude populations from developing countries and low‐ and middle‐income regions, such as China, where dementia prevalence continues to rise.^16^ Therefore, elucidating the positive impact of healthy lifestyles on cognitive function in those regions could provide valuable insights for policymakers and health administrators in addressing the growing challenge of CI and dementia.

Using data from the nationwide aging cohort (Chinese Longitudinal Healthy Longevity Survey [CLHLS]) with up to 15 years of follow‐up, our study aims to elucidate the impact of healthy lifestyles and APOE genotypes on life expectancy with and without CI among Chinese older adults.

## 2 METHODS

### 2.1 Study design and samples

The data of this study were obtained from the CLHLS, a nationally representative, ongoing longitudinal cohort study of older adults in China. Initiated in 1998, the CLHLS has conducted follow‐up surveys every 2 to 3 years, completing nine waves of national surveys until now. The study population includes participants from 22 out of 31 provinces in mainland China, representing approximately 85% of the Chinese population aged 65 and older. No proxy was used for the objective questions such as cognitive function and physical performance assessments. Detailed information on the sampling procedures and data quality has been published elsewhere.^25^ Ethical approval was obtained from the Institutional Review Board of Peking University (IRB00001052‐13074). Written informed consent was obtained from all participants or their legal representatives at baseline and during follow‐up surveys. The study was conducted according to the Declaration of Helsinki.

This longitudinal study assessed genetic data at baseline. We selected participants from the fourth wave (2008/2009) survey, who were followed up in the 2011, 2014, 2018, and 2021 waves. Our study adheres to the Strengthening the Reporting of Genetic Association Studies (STREGA) guidelines (eMethod). Among the 7980 participants with genetic testing in the fourth wave, we excluded (1) 748 participants who were either younger than 65 years or at least 100 years old, (2) 631 participants who had only one record in the database, (3) 135 participants with the ε2/ε4 genotype (because of the potential opposing effects of ε2 and ε4 alleles on Alzheimer's disease risk^10^, ^26^), and (4) 18 participants with missing data on the Mini‐Mental State Examination (MMSE) or all lifestyle indicators. Ultimately, 6448 participants aged 65 years and older met the inclusion criteria for this study. The detailed participant selection flowchart is provided in Figures A1 and A2.

### 2.2 APOE genotype

In the first six waves of the CLHLS surveys (1998, 2000, 2002, 2005, 2008/2009, and 2011/2012), saliva samples were collected from participants and sent to the Beijing Genomics Institute (BGI) for genetic testing in 2014. The study utilized a custom chip targeting approximately 27,656 SNPs associated with longevity phenotypes. Detailed information on the genetic testing project and quality control procedures is available in previous publications.^27^, ^28^ In this study, APOE genotypes were defined using the SNPs rs7412 and rs429358. Due to the small number of ε2 homozygotes (*n* = 20) and ε4 homozygotes (*n* = 49), all participants were categorized into three groups: ε3 homozygotes (ε3/ε3), ε2 carriers (ε2/ε3, ε2/ε2), and ε4 carriers (ε3/ε4, ε4/ε4). Both APOE SNPs were in Hardy–Weinberg equilibrium (*p* > .05), assessed with PLINK 1.90 beta.^29^

### 2.3 Assessment of healthy lifestyles

We considered five lifestyle factors, widely associated with cognitive function^20^, ^29^, ^30^ and memory^21^: no current smoking, no alcohol consumption, ideal diet, being physically active, and maintaining active cognitive engagement. Never smokers and those who self‐reported quitting smoking for over 10 years were considered to have a healthy lifestyle. Never drinkers and those who self‐reported abstaining from alcohol for over 10 years were considered healthy.^31^ Diet diversity was assessed using a food frequency questionnaire, recording the daily intake of various food items (fruits, vegetables, grains, meat, fish, and seafood, eggs, dairy products, legumes, sweets, garlic, nuts, and tea). Based on previous research, participants among the top 40% of the diet diversity distribution were considered healthy,^20^, ^21^ and this cut‐off value of an ideal diet in our study was defined as consuming at least five out of 12 food items daily. Being physically active was defined as self‐reported daily physical activity or participation in outdoor activities (replaced by participation in Tai Chi or square dancing from the 2018 wave onward). Participants engaging in daily physical activity or outdoor activities were considered to have a healthy lifestyle. Maintaining active cognitive engagement were defined through four questions on reading newspapers or books, playing cards or mahjong, participating in organized social activities, and visiting relatives or friends (only included in the questionnaire from the eighth wave onward), and engaging in these activities at least twice a week was considered a healthy lifestyle, similar to previous studies.^21^

**RESEARCH IN CONTEXT**

1. **Systematic review**: There are limited studies examining the effect of APOE and healthy lifestyle factors on CI risk and its related life expectancy, especially in low‐ and middle‐income areas. These studies remain insufficiently comprehensive in terms of sample size and follow‐up duration.
2. **Interpretation**: By analyzing cognitive state transitions in a large, nationwide sample of older adults in China, we identified distinct roles of different APOE genotypes in these transitions and highlighted the positive impact of a healthier lifestyle on each cognitive state transition and life expectancy. These findings are crucial for understanding how to extend life expectancy without CI in older adults and can guide policymakers and healthcare professionals in designing policies and interventions to support healthier living.
3. **Future directions**: Future studies should aim to replicate and expand upon these findings in larger East Asian populations, further examining differences in APOE gene expression across sexes and their implications for life expectancy.

We assessed the association between each lifestyle factor and cognitive function. To further investigate the relationship between lifestyle factors, APOE genotype, and CI, we categorized participants into three groups based on the number of healthy lifestyle factors: four or five healthy lifestyle factors, two or three healthy lifestyle factors, and zero or one healthy lifestyle factor.

### 2.4 Assessment of CI

Each participant underwent a cognitive function assessment in each wave of the survey. Cognitive function was evaluated using the Chinese version of the MMSE, which consists of 24 items with a total score of 30, where higher scores indicate better cognitive function. The details and validation of the Chinese version of MMSE questionnaire were confirmed in previous studies.^25^, ^32^ A MMSE score of less than 18 was considered indicative of severe CI. Additionally, participants were asked in each survey whether they had been diagnosed with dementia. In this study, CI was defined as a MMSE score of less than 18^25^, ^29^ or a self‐reported history of dementia.

### 2.5 Covariates

Sociodemographic factors, physical function, and history of diseases can influence individual cognitive function. Therefore, the key sociodemographic information in this study included age, sex (men/women), residence (urban/rural), years of schooling (never/1 to 6 years/over 6 years), living arrangement (lives alone/with family member), and marital status (currently married and living together/divorced, widowed, never married, or married but not living together). The history of disease included 13 common chronic diseases of older adults: hypertension, diabetes, heart disease, stroke, chronic respiratory diseases, tuberculosis, cataracts, glaucoma, cancer, gastric ulcer, Parkinson's disease, bedsores, and arthritis. These conditions were further categorized based on the number of diseases: none, only one, and comorbidity (two or more diseases). Disability status was assessed using the activities of daily living (ADL) questionnaire. Participants were considered disabled if they required assistance in any of the six activities: bathing, dressing, using the toilet, indoor mobility, controlling bowel and bladder, and eating. Economic factors include pension (yes/no), medical insurance (yes/no), and able to get to hospital when ill (yes/no).

### 2.6 Statistical analysis

All statistical analyses were performed using RStudio (version 4.1.2). Missing values for covariates were imputed using the nearest‐neighbor method from adjacent waves, with the proportion of missing values provided in Table A.1. To clarify the role of each risk factor and its changes during follow‐up, a three‐state continuous‐time Markov chain model with covariates was constructed (Figure A.3) to assess the hazard ratios (HRs) and 95% confidence intervals of the healthy lifestyle, APOE genotype, and their interaction among each state's transitions. In addition, we also estimated the life expectancy for different subgroups defined by the healthy lifestyle and APOE genotype. This method involved two steps. First, the continuous‐time Markov process allows for four state transitions: (1) from cognitively healthy (CH) to CI; (2) from CH to death; (3) from CI to CH; and (4) from CI to death. This multi‐state Markov model allowed transition hazards to change with age and fitted the annual transition probabilities for the four state transitions while adjusting for all covariates, including sex, year of schooling, residence, living arrangement, marital status, history of disease, and economic factors. The age dependency was modeled by computing state‐specific and marginal life expectancies using the Gompertz distribution, a commonly used parametric model for mortality rates. Second, multi‐state life tables were constructed using the annual transition probabilities, with maximum likelihood estimators as point estimates for the annual transition probabilities and 95% confidence interval estimated using 1000 bootstrap resamples. The maximum human age was set at 110 years,^33^ and the expected lifespan was calculated for individuals aged 65, 75, and 85 years. Considering the differences in life expectancy between sexes, separate models were fitted for each sex. Interaction terms between APOE genotype, healthy lifestyle, and sex in the first‐step model were used to assess the differential impacts on state transitions. The annual transition probabilities and expected lifespan were analyzed using the msm^34^ and elect packages.^35^ A two‐tailed significance level was set at *p *< .05.

Several sensitivity analyses were conducted to assess the robustness of the results. First, we used baseline data to validate the robustness of the main results. We bult a generalized linear mixed model (GLMM) to assess the association between healthy lifestyle subgroups, APOE genotype, and cognitive function. Cognitive function was included as both a binary variable (CI/CH) and a continuous variable (MMSE score) in two models, with adjustments for all covariates. A prespecified interaction term between healthy lifestyle and APOE genotype was included to consider whether the association between healthy lifestyle and CI differed according to genotype. Corrections for multiple comparisons were performed using the Benjamini–Hochberg false discovery rate (FDR) procedure,^36^ accounting for all main effects and interactions modeled. The GLMM was analyzed using the lmerTest package. Second, based on the aforementioned cross‐sectional analyses, participants who died before 2011 were excluded because an earlier death may be related to poorer physical functioning, and poorer physical functioning may have interfered with participants’ ability to maintain a healthy lifestyle. Third, in the cross‐sectional analysis, considering that previous studies^21^ suggested differences in the association of each healthy lifestyle and cognitive function, a weighted Healthy Living Index (HLI) was established and defined as the sum of the regression coefficient of each healthy lifestyle (beta) multiplied by each healthy lifestyle (binary), and the HLI was divided into three groups based on the tertile method – LOW, MID, and HIGH – and care was taken to ensure that each group had a similar number of participants. We analyzed the correlation between HLI subgroups and cognitive function to assess the robustness of the primary outcome. Fourth, considering the variability in follow‐up durations, we recalibrated life expectancy using monthly rather than yearly transition probabilities to maintain the robustness of the main results.^37^ Lastly, the life expectancy for different subgroups was re‐fitted using the Middle Riemann and Simpson methods.

## 3 RESULTS

### 3.1 Demographic characteristics

The baseline characteristics of all participants are presented in Table 1. Among the 6448 participants, 3169 were female (49.1%), with an average age of 81.9 years (SD: 9.4). A high proportion of participants had never been to school (54.6%), resided in rural areas (65.9%), and lived with family members (82.6%). Of the participants, 4386 had the APOE ε3/ε3 genotype, 913 were ε2 carriers, and 1149 were ε4 carriers. The age‐ and sex‐weighted CI rates for the three genotypes were 3.8%, 3.5%, and 5.1%, respectively. Most (63.6%) participants had two or three healthy lifestyle factors. The weighted CI prevalence for the 0–1, 2–3, and 4–5 healthy lifestyle groups were 4.4%, 4.8%, and 1.5%, respectively. More detailed information about each healthy lifestyle group can be found in Table A.2.

**TABLE 1:** Baseline characteristics of study population.

Variables	Total	ε3 homozygote	ε2 carrier	ε4 carrier
Numbers	6448	4386 (68.0)	913 (14.2)	1149 (17.8)
MMSE score	24.11 ± 7.76	24.21 ± 7.68	23.90 ± 7.89	23.89 ± 7.96
CI, yes	945 (14.7)	623 (14.2)	142 (15.6)	180 (15.7)
Weighted percentage of CI (%)	4.0	3.8	3.5	5.1
Follow‐up time, years	6.05 ± 3.98	6.06 ± 3.98	6.04 ± 4.02	6.02 ± 3.98
Death during follow‐up	4047 (62.8)	2743 (62.5)	574 (62.9)	730 (63.0)
Weighted percentage of death (%)	40.9	39.9	43.7	42.4
**Sociodemographic characteristics**				
Sex				
Men	3279 (50.9)	2222 (50.7)	456 (49.9)	601 (52.3)
Women	3169 (49.1)	2164 (49.3)	457 (50.1)	548 (47.7)
Age group				
65 to 74 years	1727 (26.8)	1186 (27.0)	226 (24.8)	315 (27.4)
75 to 84 years	1968 (30.5)	1341 (30.6)	263 (28.8)	364 (31.7)
≥85	2753 (42.7)	1859 (42.4)	424 (46.4)	470 (40.9)
Years of schooling				
Never	3520 (54.6)	2385 (54.4)	511 (56.0)	624 (54.3)
1 to 6 years	2153 (33.4)	1470 (33.5)	308 (33.7)	375 (32.6)
Over 6 years	775 (12.0)	531 (12.1)	94 (10.3)	150 (13.1)
Residence				
Urban	2198 (34.1)	1468 (33.5)	329 (36.0)	401 (34.9)
Rural	4250 (65.9)	2918 (66.5)	584 (64.0)	748 (65.1)
Living arrangement				
With family member	5328 (82.6)	3623 (82.6)	736 (80.6)	969 (84.3)
Lives alone	1120 (17.4)	763 (17.4)	177 (19.4)	180 (15.7)
Marital status				
Currently married and living together	2793 (43.3)	1893 (43.2)	385 (42.2)	515 (44.8)
Divorced, widowed, never married, or married but not living together	3655 (56.7)	2493 (56.8)	528 (57.8)	634 (55.2)
**Healthy lifestyle factors**				
Ideal dietary intake	2910 (45.1)	2030 (46.3)	408 (44.7)	472 (41.1)
Never or quit smoking at least 10 years	4143 (64.3)	2838 (64.7)	597 (65.4)	708 (61.6)
Never or quit drinking at least 10 years	4328 (67.1)	2953 (67.3)	617 (67.6)	758 (66.0)
Daily exercise	2063 (32.0)	1428 (32.6)	278 (30.4)	357 (31.1)
Active cognitive engagement	2676 (41.5)	1839 (41.9)	369 (40.4)	468 (40.7)
Numbers of heathy lifestyle factors				
0 or 1	1130 (17.5)	742 (16.9)	164 (18.0)	224 (19.5)
2 or 3	4100 (63.6)	2782 (63.4)	578 (63.3)	740 (64.4)
4 or 5	1218 (18.9)	862 (19.7)	171 (18.7)	185 (16.1)
**History of disease**				
None	2730 (42.3)	1832 (41.8)	411 (45.0)	487 (42.4)
One	2172 (33.7)	1510 (34.4)	298 (32.6)	364 (31.7)
Comorbidity	1546 (24.0)	1044 (23.8)	204 (22.3)	298 (25.9)
**Disability status, yes**	400 (6.2)	269 (6.2)	69 (7.5)	62 (5.4)
**Economic factors**				
Pension, yes	1223 (19.0)	825 (18.8)	167 (18.3)	231 (20.1)
Medical insurance, yes	4962 (77.0)	3349 (76.4)	742 (81.3)	871 (75.8)
Can get to hospital when ill, yes	5975 (92.7)	4097 (93.0)	834 (91.3)	1062 (92.4)

Abbreviations: CI: cognitive impairment; MMSE, Mini‐Mental State Examination.

### 3.2 Association of APOE genotype and healthy lifestyle on CI and its state transitions

The effects of APOE genotype, healthy lifestyle, and sociodemographic factors on cognitive state transitions in a continuous‐time Markov model are shown in Table 2. Compared to the ε3 homozygote group, ε4 carriers (HR: 1.23, 95% confidence interval: 1.01 to 1.59) had a higher risk of transitioning from a CH state to CI state over the 15‐year follow‐up, and ε2 carriers (HR: 0.88, 95% confidence interval: 0.78 to 0.98) had a reduced risk of transitioning from a CH state to death.

**TABLE 2:** Adjusted HR of APOE genotype, number of healthy lifestyle factors, and sociodemographic characteristics by each cognitive state transition in Markov chain model^*^.

Variables	CH→CI	CH→Death	CI→CH	CI→Death
APOE genotype				
ε3 homozygote	Ref.	Ref.	Ref.	Ref.
ε2 carrier	1.05 (0.84 to 1.31)	0.88 (0.79 to 0.98)^†^	0.88 (0.58 to 1.36)	0.97 (0.81 to 1.16)
ε4 carrier	1.27 (1.01 to 1.59)^†^	1.06 (0.93 to 1.21)	1.24 (0.86 to 1.78)	1.01 (0.85 to 1.21)
Sex (man)	0.75 (0.60 to 0.92)^†^	1.65 (1.46 to 1.86)^†^	1.17 (0.82 to 1.68)	1.16 (0.97 to 1.38)
Residence (urban)	0.96 (0.81 to 1.15)	0.91 (0.82 to 1.01)	0.92 (0.67 to 1.26)	1.11 (0.97 to 1.28)
Lives with spouse	1.23 (0.99 to 1.52)	1.18 (1.03 to 1.35)	1.00 (0.69 to 1.45)	1.09 (0.91 to 1.31)
Years of schooling				
Never	Ref.			
1 to 6 years	0.75 (0.60 to 0.95)^†^	0.90 (0.80 to 1.01)	1.21 (0.81 to 1.81)	1.15 (0.84 to 1.40)
Over 6 years	0.74 (0.49 to 1.12)	0.99 (0.83 to 1.18)	1.18 (0.57 to 2.45)	1.09 (0.75 to 1.59)
Healthy lifestyle factors				
0 or 1 healthy lifestyle factor	Ref.	Ref.	Ref.	Ref.
2 or 3 healthy lifestyle factors	0.90 (0.71 to 1.13)	0.76 (0.67 to 0.86)^†^	1.32 (0.86 to 2.01)	0.68 (0.57 to 0.80)^†^
4 or 5 healthy lifestyle factors	0.73 (0.54 to 0.99)^†^	0.57 (0.48 to 0.67)^†^	1.73 (1.01 to 2.99)^†^	0.37 (0.28 to 0.49)^†^

Abbreviations: APOE, apolipoprotein E; CH, cognitively healthy; CI, cognitive impairment; HR, hazard ratio.

* Adjusted age, history of disease, disability status, and economic factors.

^†^ Significance was considered to be at *p* < .05.

Compared to participants with zero to one healthy lifestyle factor, those with —four or five healthy lifestyle factors had a significantly lower risk in the transitions of CH state to CI state (HR: 0.73, 95% confidence interval: 0.54 to 0.99), CH state to death (HR: 0.57, 95% confidence interval: 0.48 to 0.67), and CI state to death (HR: 0.37, 95% confidence interval: 0.28 to 0.49), and a higher probability in CI state to CH state (HR: 1.73, 95% confidence interval: 1.01 to 2.99).

Interaction analyses of APOE genotype and healthy lifestyle subgroups are shown in Table 3. Overall, the benefits of maintaining four to five healthy lifestyles appeared to be similar across APOE genotypes, with no significant interaction between healthy lifestyles and APOE genotypes.

**TABLE 3:** Adjusted HR of APOE genotype across numbers of healthy lifestyle factors based on each cognitive state transition in Markov chain model^*^.

Analyses	CH→CI	CH→Death	CI→CH	CI→Death
APOE genotype × Healthy lifestyle factors				
ε3 homozygote × 0 or 1 healthy lifestyle factor	Ref.	Ref.	Ref.	Ref.
ε3 homozygote × 2 or 3 healthy lifestyles	0.90 (0.70 to 1.16)	0.89 (0.76 to 1.03)	1.20 (0.80 to 1.79)	0.75 (0.61 to 0.91)^†^
ε3 homozygote × 4 or 5 healthy lifestyle factors	0.69 (0.50 to 0.96)^†^	0.67 (0.55 to 0.80)^†^	1.23 (0.68 to 2.21)	0.55 (0.40 to 0.75)^†^
ε2 carrier × 0 or 1 healthy lifestyle factor	Ref.	Ref.	Ref.	Ref.
ε2 carrier × 2 or 3 healthy lifestyle factors	0.94 (0.57 to 1.54)	0.64 (0.46 to 0.79)^†^	1.02 (0.42 to 2.53)	0.97 (0.68 to 1.38)
ε2 carrier × 4 or 5 healthy lifestyle factors	0.80 (0.66 to 0.97)^†^	0.61 (0.41 to 0.92)^†^	1.16 (0.15 to 9.13)	0.55 (0.38 to 0.82)^†^
ε4 carrier × 0 or 1 healthy lifestyle factor	Ref.	Ref.	Ref.	Ref.
ε4 carrier × 2 or 3 healthy lifestyle factors	0.98 (0.57 to 1.66)	0.68 (0.51 to 0.91)^†^	1.32 (0.53 to 3.27)	0.82 (0.49 to 1.07)
ε4 carrier × 4 or 5 healthy lifestyle factors	0.88 (0.75 to 1.03)	0.60 (0.41 to 0.89)^†^	1.15 (0.30 to 4.31)	0.63 (0.36 to 1.10)
Interaction terms				
2 or 3 healthy lifestyle factors, ε2 carrier interaction	1.08 (0.63 to 1.85)	0.93 (0.65 to 1.32)	0.95 (0.36 to 2.52)	1.29 (0.87 to 1.91)
2 or 3 healthy lifestyle factors, ε4 carrier interaction	1.08 (0.60 to 1.95)	0.92 (0.67 to 1.28)	1.11 (0.69 to 3.20)	1.06 (0.81 to 1.42)
4 or 5 healthy lifestyle factors, ε2 carrier interaction	1.12 (0.28 to 4.46)	0.94 (0.58 to 1.53)	0.96 (0.43 to 2.11)	1.02 (0.33 to 2.38)
4 or 5 healthy lifestyle factors, ε4 carrier interaction	1.09 (0.51 to 2.34)	0.86 (0.42 to 1.75)	1.03 (0.28 to 3.72)	1.10 (0.72 to 1.69)

Abbreviations: APOE, apolipoprotein E; CH, cognitively healthy; CI: cognitive impairment; HR, hazard ratio.

* Adjusted sex, age, years of schooling, residence, living arrangement, marital status, history of disease, disability status, and economic factors.

^†^ Significance was considered to be at *p* < .05.

### 3.3 Sex‐specific associations of APOE genotype and healthy lifestyles with life expectancy

**FIGURE 1:**
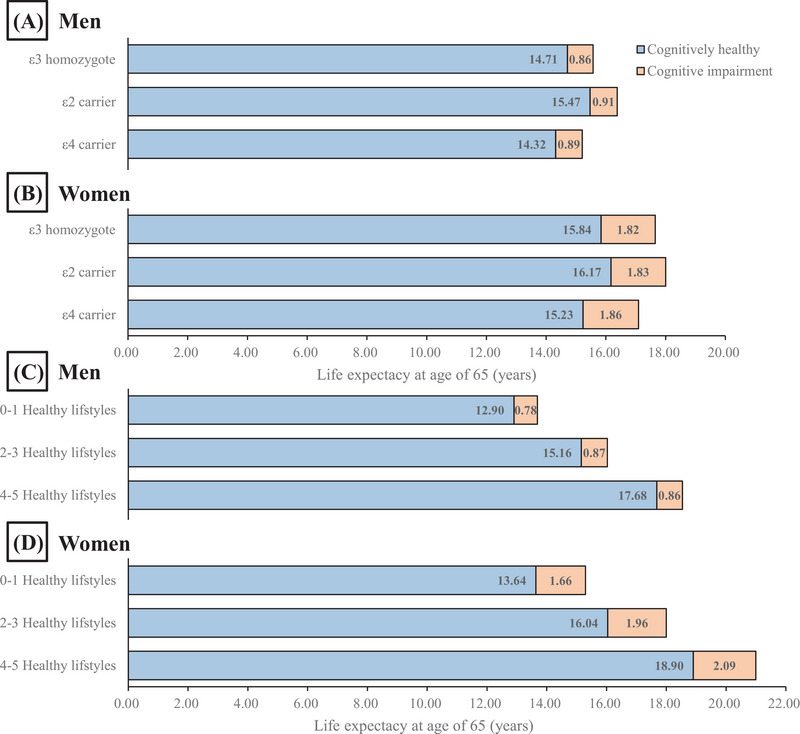
Life expectancy of study population at age 65 by different APOE genotypes and numbers of healthy lifestyle factors among both sexes: (A) men with different APOE genotype, (B) women with different APOE genotypes, (C) men with different healthy lifestyle factors, and (D) women with different healthy lifestyle factors. APOE, apolipoprotein E.

Considering the differences in life expectancy and late‐life cognition between men and women, Figure 1 presents sex‐stratified estimates of life expectancy at 65, with and without CI, for different APOE genotypes and healthy lifestyle subgroups. Women had longer life expectancies (in both cognitive states) compared to men across all analyses. ε2 carriers had a higher proportion of CH state in total life expectancy in both sexes (Table A.3).

Compared to participants with the ε3 homozygote, men with the ε2 genotype experienced increases of 0.76 years (5.2%) in CH life expectancy and 0.05 years (5.8%) in CI life expectancy, while men with the ε4 genotype had increases of −0.39 years (−2.7%) and 0.03 years (3.5%), respectively. Women with the ε2 genotype saw increases of 0.33 years (2.1%) and 0.01 years (0.5%) in cognitively healthy life expectancy and CI life expectancy, respectively, while women with the ε4 genotype showed an increase of −0.61 years (3.9%) and 0.04 years (2.2%), respectively. This pattern remained consistent at ages 75 and 85.

A greater number of healthy lifestyle factors increased both CH and CI life expectancy for both sexes. Compared to those with zero or one healthy lifestyle factor, participants with four to five healthy lifestyle factors had an increase of 4.78 years (37.0%) in CH life expectancy and 0.08 years (10.3%) in CI life expectancy for men at age 65 years; for women the increases were 5.26 years (38.6%) and 0.43 years (25.9%), respectively. Both sexes had a higher proportion of CH life expectancy, and this trend diminished with age (Table A.4).

**FIGURE 2:**
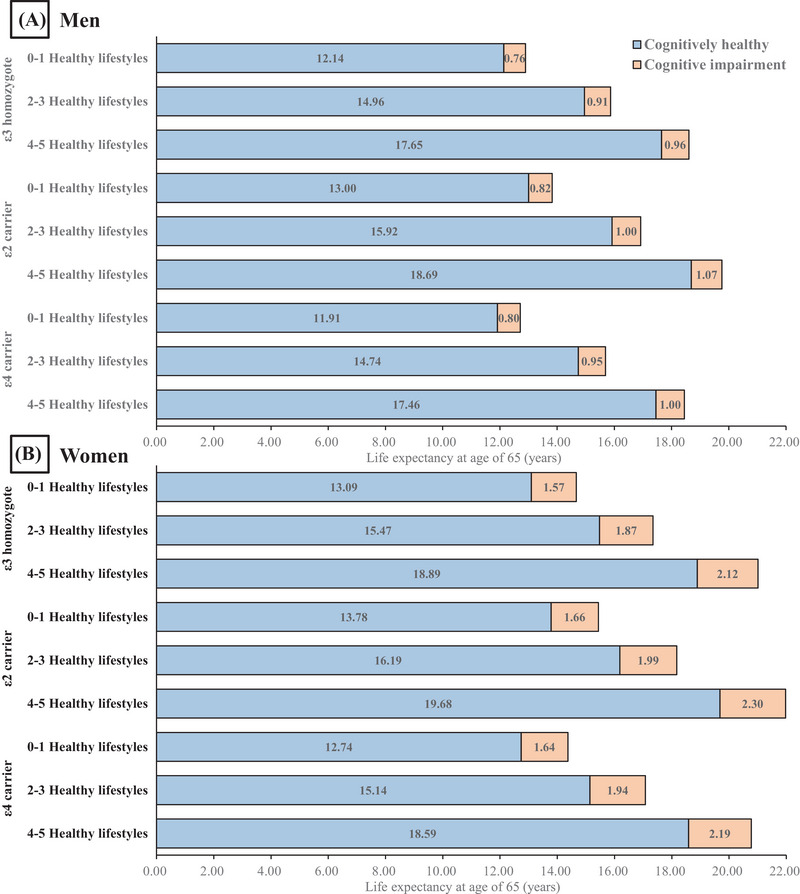
Life expectancy among APOE genotypes across different healthy lifestyle groups by cognitive status among both sexes at age 65: (A) men and (B) women. APOE, apolipoprotein E.

Figure 2 shows CH and cognitively impaired life expectancy at 65, stratified by genotype and healthy lifestyle grouping. Overall, healthy lifestyle had a higher impact on cognition‐related life expectancy than APOE genotypes and showed similar results across all APOE genotype subgroups. For men, compared to those with —zero or one healthy lifestyle factor, participants with four to five healthy lifestyle factors had an increase of 5.51 (45.3%) years in CH life expectancy and 0.20 (26.3%) years in CI life expectancy with the ε3 homozygote genotype, 5.69 (43.8%) years and 0.25 (30.5%) years with the ε2 genotype, and 5.55 (46.6%) years and 0.20 (25.0%) years with the ε4 genotype. Women with four to five healthy lifestyle factors had an increase of 5.80 (44.3%) years in CH life expectancy and 0.55 (35.0%) years in CI life expectancy for the ε3 homozygote genotype, 5.90 (42.8%) years and 0.64 (38.6%) years for the ε2 genotype, and 5.85 (45.9%) years and 0.55 (33.5%) years for the ε4 genotype. Regardless of sex and APOE genotype, more healthy lifestyle factors significantly increased CH life expectancy and its proportion (Figure A.4), though this trend diminished with age (Figure A.5 and A.6).

### 3.4 Sensitivity analysis

Sensitivity analyses were performed on all participants at baseline and by excluding participants who died within 3 years of the baseline survey. The results remained consistent with the primary findings (Tables A.5 and A.6). We also analyzed the association of each number of healthy lifestyle factors with CI and MMSE score (Table A.7). Additionally, we verified that a healthier integrated lifestyle (HLI) was associated with better cognitive functioning (Table A.8). In continuous‐time Markov models, we additionally analyzed the interaction of APOE genotype with sex, which showed no significant interaction (Table A.9). For life expectancy modeling, we used monthly transition rates (Table A.10) and different fitting methods (Table A.11 and A.12) to validate the robustness of our results.

## 4 DISCUSSION

Analyzing cognitive state transitions and life expectancy in a large, nationwide sample of older adults in China, we identified distinct roles of different APOE genotypes and lifestyle factors in these transitions. Our findings highlight the positive impact of a healthier lifestyle on each cognitive state transition. Moreover, healthy lifestyle factors outweigh the influence of APOE genetic risk on the incidence of CI and lifespan. Thus, irrespective of APOE genotype, adhering to a healthy lifestyle significantly delays the onset of CI and prolongs the CH life expectancy.

Few studies have focused on the cognitive function of APOE genotype within Chinese older people. Consistent with other studies,^29^, ^38^, ^39^, ^40^ we found that the ε4 genotype was associated with a higher risk of CH to CI than the ε3 homozygote. Our study did not find a significant difference in the risk of CI between ε2 carriers and ε3 homozygous participants. An analysis of 21,852 Asian individuals showed no association between the APOE ε2 allele and AD risk in Asian and Hispanic populations.^41^ The reasons for this discrepancy require further investigation. Interestingly, the Markov model revealed that ε2 carriers had a lower risk of transitioning from a CH state to death. This could be due to the ε2 allele's association with a reduced risk of cardiovascular disease and mortality,^42^, ^43^, ^44^ resulting in ε2 carriers having the longest healthy life expectancy and total life expectancy.

The positive impact of multiple healthy lifestyle practices on cognitive state transitions was observed consistently across the overall population and different APOE genotype subgroups in Chinese older adults, although among e4 carriers, the healthier lifestyle subgroup showed fewer significant benefits in total cognitive state transitions compared with other genotypes. Some studies have suggested that modifiable lifestyles only benefit ε4 non‐carriers,^14^ but increasing evidence supports our findings.^16^ A study involving 29,072 Chinese participants found that those adhering to a similar definition of a healthy lifestyle experienced slower memory decline, regardless of APOE ε4 carrier status.^21^ Another study based on a similar lifestyle definition in an American cohort also showed that more healthy lifestyle practices were associated with slower cognitive decline, irrespective of APOE ε4 allele presence.^45^ These findings support having an optimistic mindset with respect to the notion that maintaining a healthier lifestyle benefits delaying CI in late life, regardless of genetic risk.

By fitting life expectancy with and without CI, the effects of individual risk factors on cognitive health were observed and compared, but research estimating life expectancy for different APOE genotypes and multiple lifestyle subgroups has rarely been reported. A study in the Netherlands found that at age 65, ε4 carriers and non‐carriers had a similar life expectancy with severe CI and AD, but there were significant differences in life expectancy with cognitively healthy individuals.^46^ This aligns with our results showing higher CI risk and shorter life expectancy among ε4 carriers. Current studies on ε2 carriers indicate a correlation between the ε2 allele and longer life expectancy.^47^ Our study supported the idea that ε2 carriers have longer CH and total life expectancy and further suggests that this may benefit more from the reduced risk of death in the CH population, but the exact mechanisms need to be further investigated. Our study did not find that a healthy lifestyle could compress the years of life spent with CI, as suggested by a previous study.^20^ One reason for this discrepancy could be regional economic and lifestyle differences. For example, the ideal dietary intake threshold in our study was lower than that of participants from urban populations,^21^ despite using similar daily diet estimation methods. Importantly, there was also no evidence in our study for an expansion of this morbidity – healthy lifestyles were associated with proportional increases in both CH and cognitively impaired life expectancy.

Furthermore, our findings are consistent with the observation that genetic risk accounts for only about 5% of the variance in life expectancy, whereas a healthy lifestyle contributes to over 30% of the extension, with a higher proportion affecting CH expectancy. This suggests that a healthy lifestyle is a potential intervention for delaying or preventing CI. However, as CI life expectancy cannot be fully compressed, the absolute number of older individuals with CI may increase. This underscores the need for healthcare professionals to advocate for healthy lifestyles while also planning resource allocation to better address these challenges.

Additionally, men have a lower risk of transitioning from CH state to CI states. Some studies suggest that hormonal changes in women may pose a higher risk of cognitive decline,^8^, ^48^ but we did not observe significant sex‐specific differences in the effect of a healthy lifestyle or genetics, which suggests that the benefits of a healthy lifestyle on cognitive health are robust across both sexes. Some previous multiomics studies suggested that the APOE gene interacted with sex^49^; therefore, future research with larger, all‐age cohorts is needed to confirm sex‐related risk differences among APOE genotype in the Chinese older population.

However, our research has several limitations. First, all lifestyle and disease history data were self‐reported, introducing a risk of recall bias. Fortunately, the CLHLS questionnaire has been extensively validated,^25^ and the rates of logically inconsistent answers and incomplete data are low (1% to 3%),^50^ which can mitigate the effect of recall bias. Second, the long intervals between cognitive function assessments (2 to 4 years) add to the difficulty of capturing the differences, which at the same time may underestimate the influence of lifestyle factors. However, based on prior empirical and simulation studies, these downward biases are in large part offsetting.^51^ In addition, we used monthly transition rates for life expectancy fitting, and the robustness of the results suggests that inconsistent follow‐up intervals had a low impact on the results. Third, a healthy volunteer effect is inevitable in studies of this kind, as individuals with poor health may have refused to participate in the study. In the CLHLS survey, the interview refusal rate was only around 2%,^50^ suggesting a very small effect on healthy volunteers. Fourth, there was a contentious relationship between alcohol consumption levels and cognitive health. While some studies suggest that participants with light to moderate alcohol intake exhibit better cognitive function compared to those who abstain or rarely consume alcohol,^52^, ^53^ a plausible explanation could be the presence of reverse causation, where abstainers or non‐drinkers may have refrained due to disease or unhealthy conditions. To address this, we adopted stricter criteria for alcohol consumption. On one hand, we extended the abstinence period to 10 years, aiming to mitigate such reverse causation effects. On the other hand, some studies suggest that any level of alcohol consumption can lead to changes in brain tissue, thereby exerting an adverse impact on cognition.^31^ Lastly, not all participants underwent APOE genetic testing, and we excluded individuals with the ε2/ε4 genotype. Although we adjusted for sample weights, further research and validation are needed before generalizing the life expectancy estimates to the broader Chinese population.

## 5 CONCLUSIONS

Our study suggests that modifiable healthy lifestyle factors may delay the onset of CI in later life while extending CH life expectancy, regardless of the APOE genetic risk. Policymakers and healthcare professionals should consider implementing healthy lifestyle interventions, given the rising trend in the CI‐related burden in China and globally.

## CONFLICT OF INTEREST STATEMENT

The authors declare no conflicts of interest.

## CONSENT STATEMENT

All human subjects provided informed consent for this study.

## Supporting information



Supporting Information

Supporting Information
