# Supplementary material for: Healthy lifestyle factors outweigh influence of APOE genetic risk on extending cognitively healthy life expectancy among Chinese older adults: evidence from a nationwide cohort study
Source: Alzheimers Dement. 2025 Apr 14;21(4):e70090. doi: 10.1002/alz.70090 (PMC11995295; doi:10.1002/alz.70090)

**APPENDEX**

eMethods 2

Table A.1. Missing data for each variable of interest. 5

Table A.2. Baseline characteristic of study population divided by different lifestyle groups. 6

Table A.3. Estimated life expectancies and proportions of cognitive states among both sexes of study population at age of 65, 75 and 85 divided by different APOE genotype. 7

Table A.4. Estimated life expectancies and proportions of cognitive states among both sexes of study population at age of 65, 75 and 85 divided by different healthy lifestyle groups. 8

Table A.5. Associations of APOE genotype across sex and numbers of healthy lifestyles on cognitive function by GLMM model in baseline participants^*^. 9

Table A.6. Associations of APOE genotype and numbers of healthy lifestyles on cognitive function by GLMM model in baseline participants excluded deaths before the second survey (N=4800) ^*^. 10

Table A.7. Associations of APOE genotype and number of healthy lifestyles on cognitive function by GLMM model^*^. 11

Table A.8. Sensitivity analysis of grouping healthy lifestyle index and cognitive function by GLMM model^*^. 12

Table A.9. Adjusted hazard ratios of APOE genotype across sex on each cognitive state transitions in Markov chain model^*^. 13

Table A.10. Estimated life expectancies of cognitive states among both sexes of study population at age of 65 by monthly transition method. 14

Table A.11. Estimated life expectancies of cognitive states among both sexes of study population at age of 65 by Middle Riemann method. 15

Table A.12. Estimated life expectancies of cognitive states among both sexes of study population at age of 65 by Simpson method. 16

Figure A.1. Flow chart of the study design. 17

Figure A.2. Follow-up of the study population in each wave. 18

Figure A.3. Multistate model of transitions of cognitive states. 19

Figure A.4. Life expectancies and proportions of cognitively health and cognitive impairment state at age of 65 by different APOE genotype and healthy lifestyles among both sexes. A: Men; B: Women. 20

Figure A.5. Life expectancies and proportions of cognitively health and cognitive impairment state at age of 75 by different APOE genotype and healthy lifestyles among both sexes. A: Men; B: Women. 21

Figure A.6. Life expectancies and proportions of cognitively health and cognitive impairment state at age of 85 by different APOE genotype and healthy lifestyles among both sexes. A: Men; B: Women. 22

**eMethods**

Reporting checklist for genetic association study: Strengthening the Reporting of Genetic Association Studies ([STREGA](https://www.equator-network.org/reporting-guidelines/strobe-strega/)) guidelines.

|  |  | Reporting Item | Page Number |
| --- | --- | --- | --- |
| **Title and abstract** |  |  |  |
| Title | [#1a](https://www.goodreports.org/reporting-checklists/strega/info/#1a) | Indicate the study’s design with a commonly used term in the title or the abstract | 1 |
| Abstract | [#1b](https://www.goodreports.org/reporting-checklists/strega/info/#1b) | Provide in the abstract an informative and balanced summary of what was done and what was found | 2 |
| **Background/rationale** | [#2](https://www.goodreports.org/reporting-checklists/strega/info/#2) | Explain the scientific background and rationale for the investigation being reported | 4 |
| **Objectives** | [#3](https://www.goodreports.org/reporting-checklists/strega/info/#3) | State specific objectives, including any prespecified hypotheses. State if the study is the first report of a genetic association, a replication effort, or both. | 4 |
| **Study design** | [#4](https://www.goodreports.org/reporting-checklists/strega/info/#4) | Present key elements of study design early in the paper | 12 |
| **Setting** | [#5](https://www.goodreports.org/reporting-checklists/strega/info/#5) | Describe the setting, locations, and relevant dates, including periods of recruitment, exposure, follow-up, and data collection | 12 |
| **Eligibility criteria** | [#6a](https://www.goodreports.org/reporting-checklists/strega/info/#6a) | Cohort study – Give the eligibility criteria, and the sources and methods of selection of participants. Describe methods of follow-up. | 12 |
| **Variables** | [#7a](https://www.goodreports.org/reporting-checklists/strega/info/#7a) | Clearly define all outcomes, exposures, predictors, potential confounders, and effect modifiers. Give diagnostic criteria, if applicable | 12-15 |
|  | [#7b](https://www.goodreports.org/reporting-checklists/strega/info/#7b) | Clearly define genetic exposures (genetic variants) using a widely-used nomenclature system. Identify variables likely to be associated with population stratification (confounding by ethnic origin). | 13 |
| **Data sources/measurement** | [#8a](https://www.goodreports.org/reporting-checklists/strega/info/#8a) | For each variable of interest give sources of data and details of methods of assessment (measurement). Describe comparability of assessment methods if there is more than one group. Give information separately for for exposed and unexposed groups if applicable. | 14-16 |
|  | [#8b](https://www.goodreports.org/reporting-checklists/strega/info/#8b) | Describe laboratory methods, including source and storage of DNA, genotyping methods and platforms (including the allele calling algorithm used, and its version), error rates and call rates. State the laboratory / centre where genotyping was done. Describe comparability of laboratory methods if there is more than one group. Specify whether genotypes were assigned using all of the data from the study simultaneously or in smaller batches. | 13 |
| **Bias** | [#9a](https://www.goodreports.org/reporting-checklists/strega/info/#9a) | Describe any efforts to address potential sources of bias | 13 |
| **Study size** | [#10](https://www.goodreports.org/reporting-checklists/strega/info/#10) | Explain how the study size was arrived at | 12 |
| **Quantitative variables** | [#11](https://www.goodreports.org/reporting-checklists/strega/info/#11) | Explain how quantitative variables were handled in the analyses. If applicable, describe which groupings were chosen, and why. If applicable, describe how effects of treatment were dealt with. | 12 |
| **Statistical methods** | [#12a](https://www.goodreports.org/reporting-checklists/strega/info/#12a) | Describe all statistical methods, including those used to control for confounding. State software version used and options (or settings) chosen. | 15-16 |
|  | [#12b](https://www.goodreports.org/reporting-checklists/strega/info/#12b) | Describe any methods used to examine subgroups and interactions | 16 |
|  | [#12c](https://www.goodreports.org/reporting-checklists/strega/info/#12c) | Explain how missing data were addressed | A16-18 |
|  | [#12d](https://www.goodreports.org/reporting-checklists/strega/info/#12d) | If applicable, explain how loss to follow-up was addressed | - |
|  | [#12e](https://www.goodreports.org/reporting-checklists/strega/info/#12e) | Describe any sensitivity analyses | 16 |
|  | [#12f](https://www.goodreports.org/reporting-checklists/strega/info/#12f) | State whether Hardy-Weinberg equilibrium was considered and, if so, how. | - |
|  | [#12g](https://www.goodreports.org/reporting-checklists/strega/info/#12g) | Describe any methods used for inferring genotypes or haplotypes | 13 |
|  | [#12h](https://www.goodreports.org/reporting-checklists/strega/info/#12h) | Describe any methods used to assess or address population stratification. | 15 |
|  | [#12i](https://www.goodreports.org/reporting-checklists/strega/info/#12i) | Describe any methods used to address multiple comparisons or to control risk of false positive findings. | 15 |
|  | [#12j](https://www.goodreports.org/reporting-checklists/strega/info/#12j) | Describe any methods used to address and correct for relatedness among subjects | 15 |
| **Participants** | [#13a](https://www.goodreports.org/reporting-checklists/strega/info/#13a) | Report numbers of individuals at each stage of study—eg numbers potentially eligible, examined for eligibility, confirmed eligible, included in the study, completing follow-up, and analysed. Give information separately for for exposed and unexposed groups if applicable. Report numbers of individuals in whom genotyping was attempted and numbers of individuals in whom genotyping was successful. | A18 |
|  | [#13b](https://www.goodreports.org/reporting-checklists/strega/info/#13b) | Give reasons for non-participation at each stage | - |
|  | [#13c](https://www.goodreports.org/reporting-checklists/strega/info/#13c) | Consider use of a flow diagram | A17 |
| **Descriptive data** | [#14a](https://www.goodreports.org/reporting-checklists/strega/info/#14a) | Give characteristics of study participants (eg demographic, clinical, social) and information on exposures and potential confounders. Give information separately for exposed and unexposed groups if applicable. Consider giving information by genotype | 5 |
|  | [#14b](https://www.goodreports.org/reporting-checklists/strega/info/#14b) | Indicate number of participants with missing data for each variable of interest | A16 |
|  | [#14c](https://www.goodreports.org/reporting-checklists/strega/info/#14c) | Cohort study – Summarize follow-up time, e.g. average and total amount. | 5 |
| **Outcome data** | [#15](https://www.goodreports.org/reporting-checklists/strega/info/#15) | Cohort study Report numbers of outcome events or summary measures over time.Give information separately for exposed and unexposed groups if applicable. Report outcomes (phenotypes) for each genotype category over time | 5 |
| **Main results** | [#16a](https://www.goodreports.org/reporting-checklists/strega/info/#16a) | Give unadjusted estimates and, if applicable, confounder-adjusted estimates and their precision (eg, 95% confidence interval). Make clear which confounders were adjusted for and why they were included | 23 |
|  | [#16b](https://www.goodreports.org/reporting-checklists/strega/info/#16b) | Report category boundaries when continuous variables were categorized | 5 |
|  | [#16c](https://www.goodreports.org/reporting-checklists/strega/info/#16c) | If relevant, consider translating estimates of relative risk into absolute risk for a meaningful time period | 5 |
|  | [#16d](https://www.goodreports.org/reporting-checklists/strega/info/#16d) | Report results of any adjustments for multiple comparisons | A6 |
| **Other analyses** | [#17a](https://www.goodreports.org/reporting-checklists/strega/info/#17a) | Report other analyses done—e.g., analyses of subgroups and interactions, and sensitivity analyses | A6-15 |
| **Key results** | [#18](https://www.goodreports.org/reporting-checklists/strega/info/#18) | Summarise key results with reference to study objectives | 8 |
| **Limitations** | [#19](https://www.goodreports.org/reporting-checklists/strega/info/#19) | Discuss limitations of the study, taking into account sources of potential bias or imprecision. Discuss both direction and magnitude of any potential bias. | 10-11 |
| **Interpretation** | [#20](https://www.goodreports.org/reporting-checklists/strega/info/#20) | Give a cautious overall interpretation considering objectives, limitations, multiplicity of analyses, results from similar studies, and other relevant evidence. | 11 |
| **Generalisability** | [#21](https://www.goodreports.org/reporting-checklists/strega/info/#21) | Discuss the generalisability (external validity) of the study results | 11 |
| **Funding** | [#22](https://www.goodreports.org/reporting-checklists/strega/info/#22) | Give the source of funding and the role of the funders for the present study and, if applicable, for the original study on which the present article is based | 17 |

**Table A.1. Missing data for each variable of interest.**

| Variables | Total | Number of missing (%) |
| --- | --- | --- |
| Age | 6448 persons | 0 |
| Sex | 6448 persons | 0 |
| Residence | 16849 person-time | 0 |
| Years of schooling | 6448 persons | 0 |
| Living arrangement | 16849 person-time | 0 |
| Marital status | 16849 person-time | 15 times (0.1) |
| Ideal dietary intake | 16849 person-time | 124 times (0.7) |
| Never or quit smoking at least 10 years | 16849 person-time | 202 times (1.2) |
| Never or quit drinking at least 10 years | 16849 person-time | 253 times (1.5) |
| Daily exercise | 16849 person-time | 151 times (0.9) |
| Active cognitive engagement | 16849 person-time | 457 times (2.7) |
| History of disease | 16849 person-time | 0 |
| Disability status | 16849 person-time | 23 times (0.1) |
| Pension | 16849 person-time | 975 times (5.8) |
| Medical insurance | 16849 person-time | 975 times (5.8) |
| Able to hospital when illness | 16849 person-time | 752 times (4.5) |

**Table A.2. Baseline characteristic of study population divided by different lifestyle groups.**

| Variables | 0-1 healthy lifestyle | 2-3 healthy lifestyle | 4-5 healthy lifestyle |
| --- | --- | --- | --- |
| Numbers | 1130 | 4100 | 1218 |
| MMSE score | 23.05±8.38 | 23.72±8.03 | 26.39±5.52 |
| CI, yes | 198 (17.5) | 666 (16.2) | 81 (6.7) |
| Weighted percentage of CI, % | 4.4 | 4.8 | 1.5 |
| Follow-up time, years | 5.24±3.89 | 6.09±3.98 | 6.64±3.94 |
| Death during follow-up | 837 (74.1) | 2622 (64.0) | 588 (48.3) |
| Weighted percentage of Death, % | 53.1 | 43.3 | 26.1 |
| **Sociodemographic characteristics** |  |  |  |
| Sex |  |  |  |
| Men | 885 (78.3) | 1896 (46.2) | 498 (40.9) |
| Women | 245 (21.7) | 2204 (53.8) | 720 (59.1) |
| Age group |  |  |  |
| 65-74 years | 240 (21.2) | 1079 (26.3) | 408 (33.5) |
| 75-84 years | 357 (31.6) | 1249 (30.5) | 362 (29.7) |
| 85 and over | 533 (47.2) | 1772 (43.2) | 448 (36.8) |
| Years of schooling |  |  |  |
| Never | 560 (49.6) | 2418 (59.0) | 542 (44.5) |
| 1-6 years | 462 (40.9) | 1249 (30.5) | 442 (36.3) |
| Over 6 years | 108 (9.6) | 433 (10.6) | 234 (19.2) |
| Residence |  |  |  |
| Urban | 290 (25.7) | 1246 (30.4) | 662 (54.4) |
| Rural | 840 (74.3) | 2854 (69.6) | 556 (45.6) |
| Living arrangement |  |  |  |
| With family member | 920 (81.4) | 3382 (82.5) | 1026 (84.2) |
| Lives alone | 210 (18.6) | 718 (17.5) | 192 (15.8) |
| Marital status |  |  |  |
| Currently married and live together | 513 (45.4) | 1686 (41.1) | 594 (48.8) |
| Divorced, widowed, never married, or married but not live together | 617 (54.6) | 2414 (58.9) | 624 (51.2) |
| **History of disease** |  |  |  |
| None | 538 (47.6) | 1735 (42.3) | 457 (37.5) |
| One | 360 (31.9) | 1417 (34.6) | 395 (32.4) |
| Comorbidity | 232 (20.5) | 948 (23.1) | 366 (30.0) |
| **Disability status, yes** | 90 (8.0) | 286 (7.0) | 24 (2.0) |
| **Economic factors** |  |  |  |
| Pension, yes | 127 (11.2) | 650 (15.9) | 446 (36.6) |
| Medical insurance, yes | 836 (74.0) | 3179 (77.5) | 947 (77.8) |
| Able to hospital when illness, yes | 1008 (89.2) | 3777 (92.1) | 1190 (97.7) |

Abbreviations: CI: Cognitive impairment.

**Table A.3. Estimated life expectancies and proportions of cognitive states among both sexes of study population at age of 65, 75 and 85 divided by different APOE genotype.**

|  | ε3 homozygote | ε2 carrier | ε4 carrier |
| --- | --- | --- | --- |
| At age of 65 years |  |  |  |
| Men |  |  |  |
| Total LE | 15.569 (15.062-16.044) | 16.380 (15.521-17.192) | 15.211 (14.474-15.919) |
| CH LE | 14.713 (14.235-15.168) | 15.472 (14.696-16.259) | 14.317 (13.641-14.999) |
| % of Total LE | 94.50 | 94.52 | 94.12 |
| CI LE | 0.857 (0.761-0.958) | 0.908 (0.760-1.078) | 0.894 (0.760-1.052) |
| % of Total LE | 5.50 | 5.48 | 5.88 |
| Women |  |  |  |
| Total LE | 17.655 (17.134-18.162) | 18.000 (17.213-18.737) | 17.083 (16.406-17.745) |
| CH LE | 15.840 (15.355-16.318) | 16.169 (15.411-16.895) | 15.233 (14.532-15.862) |
| % of Total LE | 89.72 | 89.83 | 89.17 |
| CI LE | 1.815 (1.635-2.007) | 1.831 (1.561-2.137) | 1.860 (1.601-2.146) |
| % of Total LE | 10.28 | 10.17 | 10.83 |
| At age of 75 years |  |  |  |
| Men |  |  |  |
| Total LE | 9.532 (9.224-9.821) | 10.136 (9.553-10.712) | 9.255 (8.756-9.732) |
| CH LE | 8.769 (8.486-9.068) | 9.328 (8.780-9.894) | 8.459 (8.007-8.920) |
| % of Total LE | 91.99 | 92.03 | 91.39 |
| CI LE | 0.763 (0.688-0.844) | 0.808 (0.669-0.958) | 0.796 (0.675-0.930) |
| % of Total LE | 8.01 | 7.97 | 8.60 |
| Women |  |  |  |
| Total LE | 11.120 (10.774-11.463) | 11.384 (10.829-11.933) | 10.681 (10.192-11.163) |
| CH LE | 9.495 (9.181-9.795) | 9.746 (9.220-10.281) | 9.019 (8.568-9.469) |
| % of Total LE | 85.38 | 85.61 | 84.44 |
| CI LE | 1.626 (1.489-1.770) | 1.637 (1.407-1.893) | 1.662 (1.443-1.895) |
| % of Total LE | 14.61 | 14.39 | 15.56 |
| At age of 85 years |  |  |  |
| Men |  |  |  |
| Total LE | 5.235 (5.050-5.422) | 5.615 (5.254-6.002) | 5.054 (4.757-5.366) |
| CH LE | 4.567 (4.397-4.742) | 4.907 (4.577-5.250) | 4.357 (4.090-4.636) |
| % of Total LE | 87.24 | 87.39 | 86.21 |
| CI LE | 0.668 (0.596-0.739) | 0.707 (0.590-0.834) | 0.697 (0.589-0.815) |
| % of Total LE | 12.76 | 12.61 | 13.79 |
| Women |  |  |  |
| Total LE | 6.327 (6.121-6.540) | 6.494 (6.138-6.868) | 6.046 (5.729-6.375) |
| CH LE | 4.929 (4.739-5.121) | 5.084 (4.783-5.439) | 4.617 (4.339-4.920) |
| % of Total LE | 77.90 | 78.29 | 76.36 |
| CI LE | 1.398 (1.284-1.511) | 1.410 (1.211-1.626) | 1.429 (1.238-1.630) |
| % of Total LE | 55.10 | 21.71 | 23.64 |

Abbreviations: LE: life expectancy; CH: cognitively health; CI: cognitive impairment.

**Table A.4. Estimated life expectancies and proportions of cognitive states among both sexes of study population at age of 65, 75 and 85 divided by different healthy lifestyle groups.**

|  | 0-1 healthy lifestyle | 2-3 healthy lifestyle | 4-5 healthy lifestyle |
| --- | --- | --- | --- |
| At age of 65 years |  |  |  |
| Men |  |  |  |
| Total LE | 13.685 (13.159-14.206) | 16.025 (15.543-16.462) | 18.540 (17.767-19.316) |
| CH LE | 12.901 (12.397-13.409) | 15.155 (14.707-15.586） | 17.678 (17.003-18.365) |
| % of Total LE | 94.27 | 94.57 | 95.35 |
| CI LE | 0.784 (0.678-0.905) | 0.870 (0.780-0.965) | 0.862 (0.714-1.021) |
| % of Total LE | 5.73 | 5.43 | 4.65 |
| Women |  |  |  |
| Total LE | 15.296 (14.679-15.875) | 17.999 (17.486-18.464) | 20.992 (20.273-21.679) |
| CH LE | 13.637 (13.035-14.190) | 16.042 (15.566-16.485) | 18.904 (18.274-19.523) |
| % of Total LE | 89.15 | 89.13 | 90.05 |
| CI LE | 1.659 (1.426-1.924) | 1.957 (1.783-2.159) | 2.088 (1.846-2.373) |
| % of Total LE | 10.85 | 10.87 | 9.95 |
| At age of 75 years |  |  |  |
| Men |  |  |  |
| Total LE | 8.002 (7.681-8.330) | 9.712 (9.446-9.981) | 11.702 (11.172-12.274) |
| CH LE | 7.331 (7.015-7.653) | 8.878 (8.630-9.145) | 10.817 (10.332-11.310) |
| % of Total LE | 91.61 | 91.41 | 92.44 |
| CI LE | 0.671 (0.588-0.766) | 0.834 (0.755-0.920) | 0.885 (0.745-1.042) |
| % of Total LE | 8.39 | 8.59 | 7.56 |
| Women |  |  |  |
| Total LE | 9.154 (8.763-9.528) | 11.145 (10.827-11.449) | 13.571 (13.043-14.067) |
| CH LE | 7.768 (7.367-8.152) | 9.344 (9.037-9.623) | 11.509 (11.023-11.977) |
| % of Total LE | 84.86 | 83.84 | 84.81 |
| CI LE | 1.385 (1.197-1.601) | 1.801 (1.663-1.958) | 2.062 (1.834-2.303) |
| % of Total LE | 15.14 | 16.16 | 15.19 |
| At age of 85 years |  |  |  |
| Men |  |  |  |
| Total LE | 4.186 (4.002-4.397) | 5.250 (5.076-5.423) | 6.596 (6.258-6.978) |
| CH LE | 3.677 (3.491-3.885) | 4.527 (4.367-4.691) | 5.724 (5.435-6.055) |
| % of Total LE | 87.84 | 86.23 | 86.78 |
| CI LE | 0.509 (0.443-0.585) | 0.723 (0.649-0.797) | 0.871 (0.730-1.022) |
| % of Total LE | 12.16 | 13.77 | 13.22 |
| Women |  |  |  |
| Total LE | 4.935 (4.693-5.165) | 6.194 (6.007-6.378) | 7.860 (7.492-8.200) |
| CH LE | 3.910 (3.668-4.160) | 4.718 (4.552-4.889) | 5.970 (5.660-6.286) |
| % of Total LE | 79.23 | 76.17 | 75.95 |
| CI LE | 1.025 (0.884-1.174) | 1.476 (1.372-1.583) | 1.890 (1.665-2.117) |
| % of Total LE | 20.77 | 23.83 | 24.05 |

Abbreviations: LE: life expectancy; CH: cognitively health; CI: cognitive impairment.

**Table A.5. Associations of APOE genotype across sex and numbers of healthy lifestyles on cognitive function by GLMM model in baseline participants^*^.**

|  | Cognitive impairment | |  | MMSE score | |
| --- | --- | --- | --- | --- | --- |
|  | Adjusted OR (95%CI) | *p* |  | Adjusted Beta (se) | *p* |
| APOE genotype |  |  |  |  |  |
| ε3 homozygote | Ref. |  |  | Ref. |  |
| ε2 carrier | 0.83 (0.57-1.22) | 0.342 |  | 0.06 (0.17) | 0.708 |
| ε4 carrier | 1.45 (1.09-1.93) | 0.011 |  | -0.29 (0.15) | 0.049 |
| Healthy lifestyles |  |  |  |  |  |
| 0-1 healthy lifestyles | Ref. |  |  | Ref. |  |
| 2-3 healthy lifestyles | 1.09 (0.78-1.54) | 0.611 |  | 0.12 (0.16) | 0.443 |
| 4-5 healthy lifestyles | 0.55 (0.33-0.91) | 0.021 |  | 1.37 (0.19) | <0.001 |
| APOE genotype * Sex |  |  |  |  |  |
| ε3 homozygote * Men | Ref. |  |  | Ref. |  |
| ε2 carrier * Men | 0.94 (0.51-1.75) | 0.849 |  | 0.05 (0.19) | 0.791 |
| ε4 carrier * Men | 1.67 (1.09-2.56) | 0.017^†^ |  | -0.50 (0.16) | 0.002^†^ |
| ε3 homozygote * Women | Ref. |  |  |  |  |
| ε2 carrier * Women | 0.84 (0.53-1.34) | 0.468 |  | 0.38 (0.24) | 0.108 |
| ε4 carrier * Women | 1.25 (0.87-1.80) | 0.235 |  | -0.09 (0.21) | 0.671 |
| ε2 carrier, sex interaction | 0.99 (0.44-2.25) | 0.988 |  | 0.32 (0.30) | 0.299 |
| ε4 carrier, sex interaction | 1.22 (0.68-2.19) | 0.513 |  | 0.40 (0.26) | 0.121 |
| APOE genotype * Healthy lifestyles |  |  |  |  |  |
| ε3 homozygote * 0-1 healthy lifestyles | Ref. |  |  | Ref. |  |
| ε3 homozygote * 2-3 healthy lifestyles | 1.25 (0.83-1.89) | 0.287 |  | 0.03 (0.18) | 0.856 |
| ε3 homozygote * 4-5 healthy lifestyles | 0.45 (0.25-0.83) | 0.010 ^†^ |  | 0.82 (0.22) | <0.001^†^ |
| ε2 carrier * 0-1 healthy lifestyles | Ref. |  |  | Ref. |  |
| ε2 carrier * 2-3 healthy lifestyles | 0.46 (0.22-0.97) | 0.042 |  | 0.42 (0.42) | 0.318 |
| ε2 carrier * 4-5 healthy lifestyles | 0.04 (0.01-0.50) | 0.012 ^†^ |  | 1.16 (0.48) | 0.016^†^ |
| ε4 carrier * 0-1 healthy lifestyles | Ref. |  |  | Ref. |  |
| ε4 carrier * 2-3 healthy lifestyles | 1.11 (0.60-2.06) | 0.743 |  | 0.46 (0.30) | 0.130 |
| ε4 carrier * 4-5 healthy lifestyles | 0.63 (0.26-1.55) | 0.316 |  | 0.95 (0.38) | 0.012^†^ |
| 2-3 healthy lifestyles, ε2 carrier interaction | 0.40 (0.16-1.00) | 0.049 |  | 0.38 (0.45) | 0.395 |
| 2-3 healthy lifestyles, ε4 carrier interaction | 0.89 (0.42-1.92) | 0.771 |  | 0.43 (0.35) | 0.222 |
| 4-5 healthy lifestyles, ε2 carrier interaction | 0.10 (0.01-1.26) | 0.075 |  | 0.33 (0.51) | 0.516 |
| 4-5 healthy lifestyles, ε4 carrier interaction | 1.54 (0.53-4.51) | 0.431 |  | 0.13 (0.42) | 0.759 |

^*^: Both model adjusted sex, age, years of schooling, residence, living arrangement, marital status, history of disease, disability status, and economic factors.

^†^: Statistical differences remained after FDR correction.

**Table A.6. Associations of APOE genotype and numbers of healthy lifestyles on cognitive function by GLMM model in baseline participants excluded deaths before the second survey (N=4800) ^*^.**

|  | Cognitive impairment | |  | MMSE score | |
| --- | --- | --- | --- | --- | --- |
|  | Adjusted OR (95%CI) | *p* |  | Adjusted Beta (se) | *p* |
| APOE genotype |  |  |  |  |  |
| ε3 homozygote | Ref. |  |  | Ref. |  |
| ε2 carrier | 0.584 (0.353-0.981) | 0.042 |  | 0.285 (0.156) | 0.069 |
| ε4 carrier | 1.414 (1.001-1.996) | 0.049 |  | -0.230 (0.115) | 0.040 |
| Numbers of healthy lifestyles |  |  |  |  |  |
| 0-1 | Ref. |  |  | Ref. |  |
| 2-3 | 1.349 (0.843-2.159) | 0.212 |  | 0.287 (0.169) | 0.089 |
| 4-5 | 0.530 (0.287-0.980) | 0.043 |  | 1.222 (0.202) | <0.001 |
| APOE genotype * Sex |  |  |  |  |  |
| ε3 homozygote * Men | Ref. |  |  | Ref. |  |
| ε2 carrier * Men | 0.392 (0.102-1.507) | 0.173 |  | 0.048 (0.194) | 0.806 |
| ε4 carrier * Men | 1.511 (1.044-2.187) | 0.029^†^ |  | -0.392 (0.163) | 0.016^†^ |
| ε3 homozygote * Women | Ref. |  |  | Ref. |  |
| ε2 carrier * Women | 0.669 (0.383-1.169) | 0.158 |  | 0.021 (0.217) | 0.924 |
| ε4 carrier * Women | 1.233 (0.815-1.867) | 0.322 |  | -0.501 (0.245) | 0.041 |
| APOE genotype * Healthy lifestyles |  |  |  |  |  |
| ε3 homozygote * 0-1 healthy lifestyles | Ref. |  |  | Ref. |  |
| ε3 homozygote * 2-3 healthy lifestyles | 1.050 (0.565-1.951) | 0.877 |  | -0.075 (0.486) | 0.878 |
| ε3 homozygote * 4-5 healthy lifestyles | 0.224 (0.063-0.792) | 0.020^†^ |  | 1.876 (0.785) | 0.017^†^ |
| ε2 carrier * 0-1 healthy lifestyles | Ref. |  |  | Ref. |  |
| ε2 carrier * 2-3 healthy lifestyles | 0.923 (0.542) | 0.088 |  | 0.011 (1.019) | 0.991 |
| ε2 carrier * 4-5 healthy lifestyles | - ^‡^ | - |  | 4.304 (1.640) | 0.009^†^ |
| ε4 carrier * 0-1 healthy lifestyles | Ref. |  |  | Ref. |  |
| ε4 carrier * 2-3 healthy lifestyles | 0.783 (0.322-1.904) | 0.590 |  | 0.935 (1.064) | 0.380 |
| ε4 carrier * 4-5 healthy lifestyles | 0.178 (0.026-1.195) | 0.076 |  | 1.253 (0.670) | 0.061 |

^*^: Both model adjusted sex, age, years of schooling, residence, living arrangement, marital status, history of disease, disability status, and economic factors.

^†^: Statistical differences remained after FDR correction.

^‡^: Number of group participants less than 100 participants.

**Table A.7. Associations of APOE genotype and number of healthy lifestyles on cognitive function by GLMM model^*^.**

|  | Cognitive impairment | |  | MMSE score | |
| --- | --- | --- | --- | --- | --- |
|  | Adjusted OR (95%CI) | *p* |  | Adjusted Beta (se) | *p* |
| **APOE genotype** |  |  |  |  |  |
| ε3 homozygote | Ref. |  |  | Ref. |  |
| ε2 carrier | 0.800 (0.543-1.179) | 0.260 |  | 0.168 (0.163) | 0.303 |
| ε4 carrier | 1.451 (1.085-1.940) | 0.012 |  | -0.283 (0.140) | 0.043 |
| **Number of healthy lifestyles** |  |  |  |  |  |
| 0 | Ref. |  |  | Ref. |  |
| 1 | 0.643 (0.398-1.040) | 0.072 |  | 1.213 (0.313) | <0.001 |
| 2 | 0.347 (0.197-0.611) | <0.001 |  | 1.513 (0.332) | <0.001 |
| 3 | 0.325 (0.194-0.544) | <0.001 |  | 1.880 (0.316) | <0.001 |
| 4 | 0.197 (0.106-0.368) | <0.001 |  | 2.553 (0.333) | <0.001 |
| 5 | 0.086 (0.026-0.288) | <0.001 |  | 2.545 (0.390) | <0.001 |

^*^: Both model adjusted sex, age, years of schooling, residence, living arrangement, marital status, history of disease, disability status, and economic factors.

**Table A.8. Sensitivity analysis of grouping healthy lifestyle index and cognitive function by GLMM model^*^.**

|  | Cognitive impairment | |  | MMSE score | |
| --- | --- | --- | --- | --- | --- |
|  | Adjusted OR (95%CI) | *p* |  | Adjusted Beta (se) | *p* |
| **Healthy lifestyles index groups** |  |  |  |  |  |
| Low | Ref. |  |  | Ref. |  |
| Mid | 0.450 (0.344-0.589) | <0.001 |  | 1.308 (0.135) | <0.001 |
| High | 0.240 (0.156-0.369) | <0.001 |  | 1.717 (0.153) | <0.001 |

^*^: Both model adjusted APOE genotype, sex, age, years of schooling, residence, living arrangement, marital status, history of disease, disability status, and economic factors.

**Table A.9. Adjusted hazard ratios of APOE genotype across sex on each cognitive state transitions in Markov chain model^*^.**

|  | CH→CI | CH→Death | CI→CH | CI→Death |
| --- | --- | --- | --- | --- |
| APOE genotype * Sex |  |  |  |  |
| ε3 homozygote * Men | Ref. | Ref. | Ref. | Ref. |
| ε2 carrier * Men | 0.93 (0.66-1.31) | 0.83 (0.69-1.00) ^†^ | 0.91 (0.49-1.69) | 1.00 (0.75-1.34) |
| ε4 carrier * Men | 1.36 (1.00-1.84) ^†^ | 1.02 (0.87-1.20) | 1.14 (0.67-1.94) | 1.34 (1.03-1.75) ^†^ |
| ε3 homozygote * Women | Ref. | Ref. | Ref. | Ref. |
| ε2 carrier * Women | 1.04 (0.79-1.39) | 0.77 (0.58-1.00) ^†^ | 0.98 (0.57-1.67) | 1.02 (0.81-1.28) |
| ε4 carrier * Women | 1.23 (1.01-1.50) ^†^ | 1.09 (0.87-1.36) | 1.13 (0.70-1.84) | 0.82 (0.65-1.05) |
| ε2 carrier, sex interaction | 1.04 (0.67-1.61) | 0.90 (0.53-1.23) | 1.01 (0.46-2.19) | 1.03 (0.73-1.47) |
| ε4 carrier, sex interaction | 0.95 (0.73-1.37) | 1.02 (0.51-2.05) | 0.97 (0.74-1.26) | 0.92 (0.65-1.29) |

^*^: Adjusted sex, age, years of schooling, residence, living arrangement, marital status, history of disease, disability status, and economic factors.

^†^: Significance was considered at P < 0.05.

Abbreviations: CH: cognitively health; CI: cognitive impairment.

**Table A.10. Estimated life expectancies of cognitive states among both sexes of study population at age of 65 by monthly transition method.**

|  | CH LE | CI LE | Total LE |
| --- | --- | --- | --- |
| Men |  |  |  |
| APOE genotype |  |  |  |
| ε3 homozygote | 14.636 (14.150-15.106) | 0.847 (0.750-0.951) | 15.482 (14.972-15.965) |
| ε2 carrier | 15.249 (14.481-16.042) | 0.899 (0.754-1.075) | 16.149 (15.282-16.964) |
| ε4 carrier | 14.089 (13.424-14.761) | 0.883 (0.747-1.041) | 14.972 (14.260-15.657) |
| Healthy lifestyles |  |  |  |
| 0-1 healthy lifestyles | 12.210 (11.719-12.720) | 0.776 (0.667-0.899) | 12.987 (12.470-13.503) |
| 2-3 healthy lifestyles | 14.938 (14.491-15.390) | 0.942 (0.840-1.047) | 15.879 (15.384-16.327) |
| 4-5 healthy lifestyles | 18.518 (17.800-19.248) | 1.075 (0.892-1.265) | 19.593 (18.789-17.111) |
| Women |  |  |  |
| APOE genotype |  |  |  |
| ε3 homozygote | 15.777 (15.278-16.261) | 1.810 (1.622-2.010) | 17.587 (17.050-18.102) |
| ε2 carrier | 16.130 (15.389-16.835) | 1.853 (1.582-1.845) | 17.983 (17.193-18.702) |
| ε4 carrier | 15.087 (14.428-15.701) | 1.875 (1.609-2.165) | 16.961 (16.278-17.614) |
| Healthy lifestyles |  |  |  |
| 0-1 healthy lifestyles | 13.082 (11.526-12.646) | 1.581 (1.239-1.713) | 14.663 (12.974-14.161) |
| 2-3 healthy lifestyles | 15.208 (14.727-15.671) | 1.868 (1.687-2.068) | 17.076 (16.541-17.561) |
| 4-5 healthy lifestyles | 18.895 (18.222-19.578) | 2.197 (1.928-2.491) | 21.092 (20.354-21.822) |

Abbreviations: LE: life expectancy; CH: cognitively health; CI: cognitive impairment.

**Table A.11. Estimated life expectancies of cognitive states among both sexes of study population at age of 65 by Middle Riemann method.**

|  | CH LE | CI LE | Total LE |
| --- | --- | --- | --- |
| Men |  |  |  |
| APOE genotype |  |  |  |
| ε3 homozygote | 14.679 (14.201-15.133) | 0.856 (0.761-0.957) | 15.535 (15.028-16.009) |
| ε2 carrier | 15.437 (14.662-16.224) | 0.908 (0.760-1.077) | 16.345 (15.487-17.155) |
| ε4 carrier | 14.283 (13.609-14.964) | 0.894 (0.760-1.051) | 15.177 (14.441-15.883) |
| Healthy lifestyles |  |  |  |
| 0-1 healthy lifestyles | 12.868 (12.366-13.374) | 0.783 (0.678-0.904) | 13.651 (13.127-14.172) |
| 2-3 healthy lifestyles | 15.119 (14.672-15.549) | 0.870 (0.780-0.965) | 15.989 (15.508-16.425) |
| 4-5 healthy lifestyles | 17.640 (16.965-18.327) | 0.862 (0.714-1.021) | 18.502 (17.730-19.278) |
| Women |  |  |  |
| APOE genotype |  |  |  |
| ε3 homozygote | 15.804 (15.319-16.281) | 1.815 (1.634-2.006) | 17.619 (17.098-18.125) |
| ε2 carrier | 16.133 (15.376-16.858) | 1.830 (1.560-2.136) | 17.963 (17.177-18.698) |
| ε4 carrier | 15.187 (14.497-15.825) | 1.859 (1.601-2.145) | 17.046 (16.371-17.707) |
| Healthy lifestyles |  |  |  |
| 0-1 healthy lifestyles | 13.603 (13.001-14.154) | 1.658 (1.426-1.923) | 15.261 (14.645-15.839) |
| 2-3 healthy lifestyles | 16.004 (15.529-16.446) | 1.957 (1.783-2.158) | 17.960 (17.448-18.425) |
| 4-5 healthy lifestyles | 18.863 (18.235-19.482) | 2.088 (1.846-2.373) | 20.951 (20.233-21.638) |

Abbreviations: LE: life expectancy; CH: cognitively health; CI: cognitive impairment.

**Table A.12. Estimated life expectancies of cognitive states among both sexes of study population at age of 65 by Simpson method.**

|  | CH LE | CI LE | Total LE |
| --- | --- | --- | --- |
| Men |  |  |  |
| APOE genotype |  |  |  |
| ε3 homozygote | 14.663 (14.186-15.119) | 0.856 (0.761-0.947) | 15.519 (15.013-15.994) |
| ε2 carrier | 15.427 (14.647-16.210) | 0.907 (0.759-1.077) | 16.330 (15.471-17.142) |
| ε4 carrier | 14.268 (13.592-14.950) | 0.894 (0.759-1.051) | 15.161 (14.424-15.869) |
| Healthy lifestyles |  |  |  |
| 0-1 healthy lifestyles | 12.852 (12.348-13.359) | 0.783 (0.677-0.904) | 13.635 (13.110-14.156) |
| 2-3 healthy lifestyles | 15.106 (14.658-15.537) | 0.869 (0.780-0.965) | 15.975 (15.493-16.412) |
| 4-5 healthy lifestyles | 17.628 (16.954-18.316) | 0.861 (0.714-1.020) | 18.490 (17.717-19.266) |
| Women |  |  |  |
| APOE genotype |  |  |  |
| ε3 homozygote | 15.791 (15.305-16.268) | 1.815 (1.634-2.006) | 17.605 (17.084-18.113) |
| ε2 carrier | 16.120 (15.362-16.845) | 1.830 (1.560-2.136) | 17.950 (17.163-18.687) |
| ε4 carrier | 15.174 (14.482-15.813) | 1.859 (1.601-2.146) | 17.033 (16.356-17.695) |
| Healthy lifestyles |  |  |  |
| 0-1 healthy lifestyles | 13.588 (12.985-14.141) | 1.658 (1.426-1.923) | 15.246 (14.629-15.825) |
| 2-3 healthy lifestyles | 15.993 (15.517-16.436) | 1.956 (1.782-2.158) | 17.949 (17.436-18.414) |
| 4-5 healthy lifestyles | 18.855 (18.225-19.474) | 2.087 (1.846-2.373) | 20.942 (20.223-21.629) |

Abbreviations: LE: life expectancy; CH: cognitively health; CI: cognitive impairment.

**
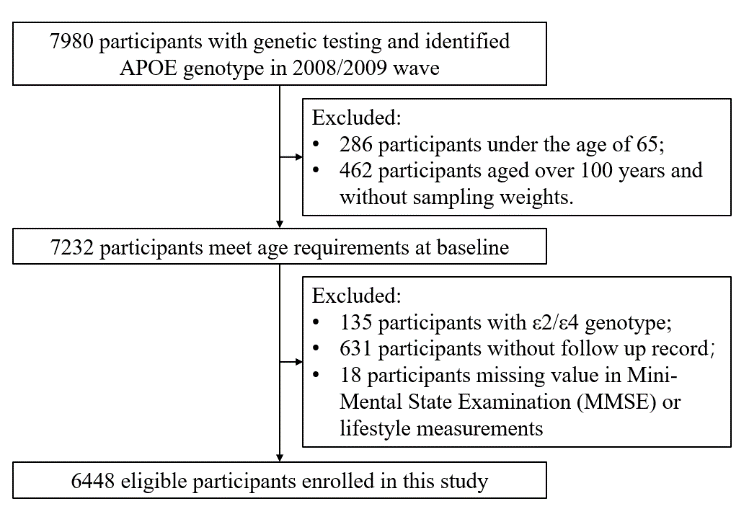
Figure A.1. Flow chart of the study design.**

**
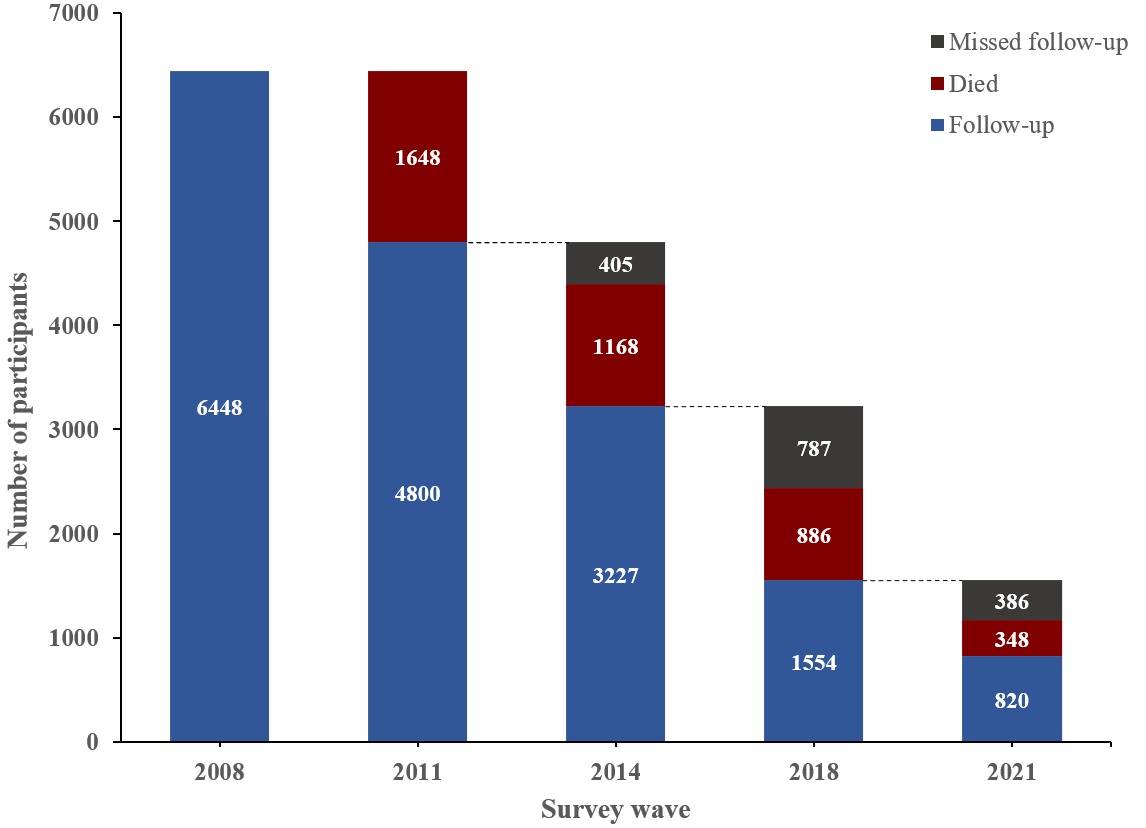
Figure A.2. Follow-up of the study population in each wave.**

**Figure A.3. Multistate model of transitions of cognitive states.**


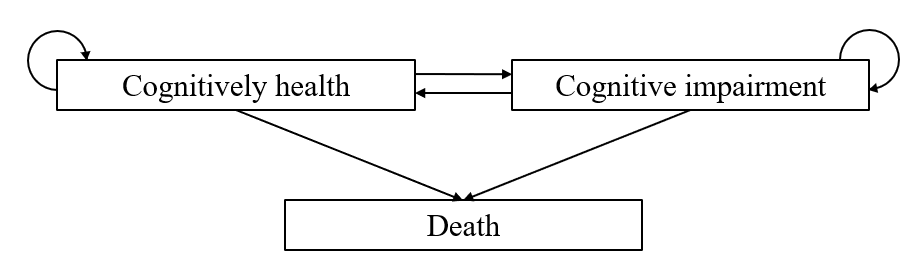


**Figure A.4. Life expectancies and proportions of cognitively health and cognitive impairment state at age of 65 by different APOE genotype and healthy lifestyles among both sexes. A: Men; B: Women.**


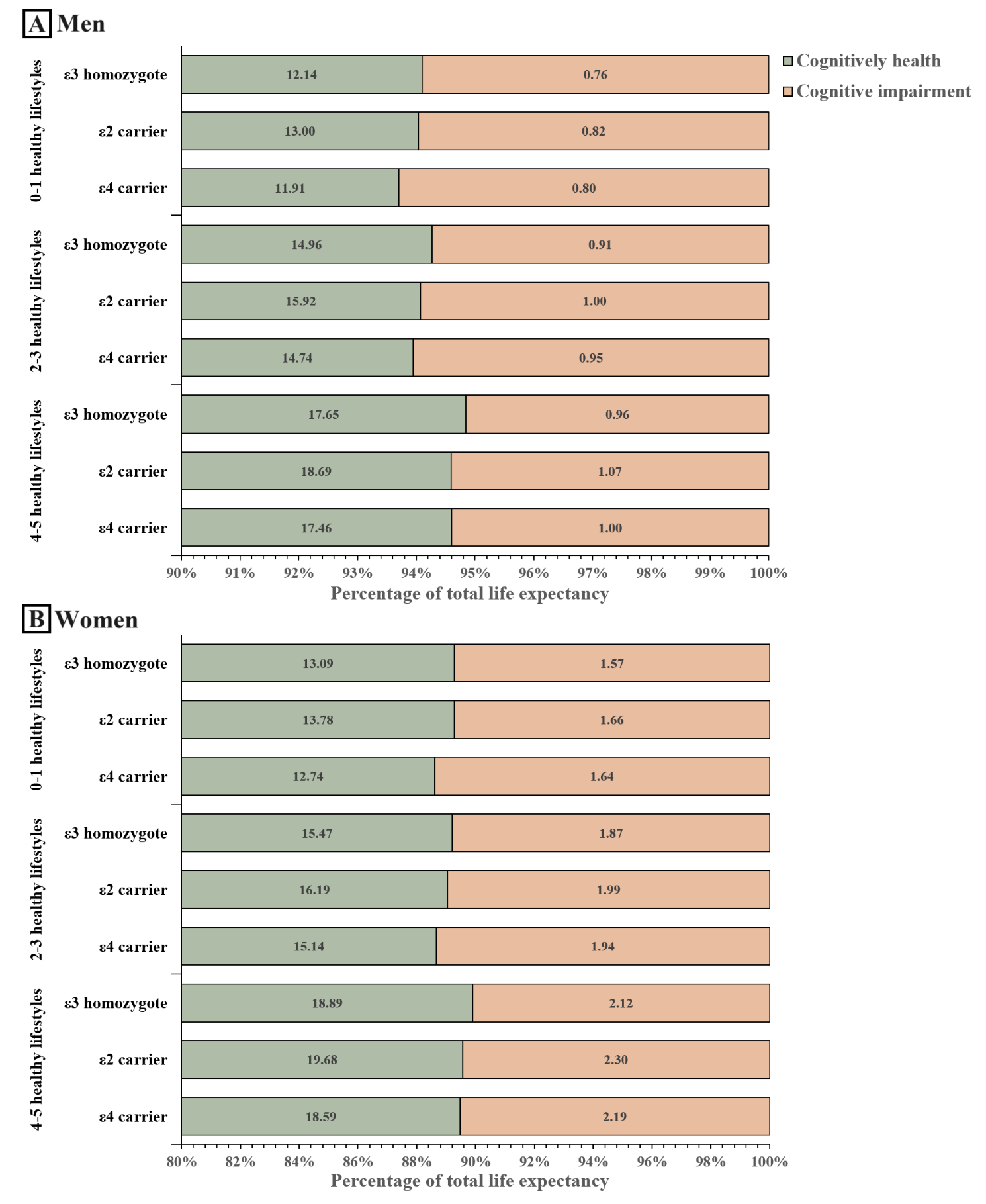


**Figure A.5. Life expectancies and proportions of cognitively health and cognitive impairment state at age of 75 by different APOE genotype and healthy lifestyles among both sexes. A: Men; B: Women.**


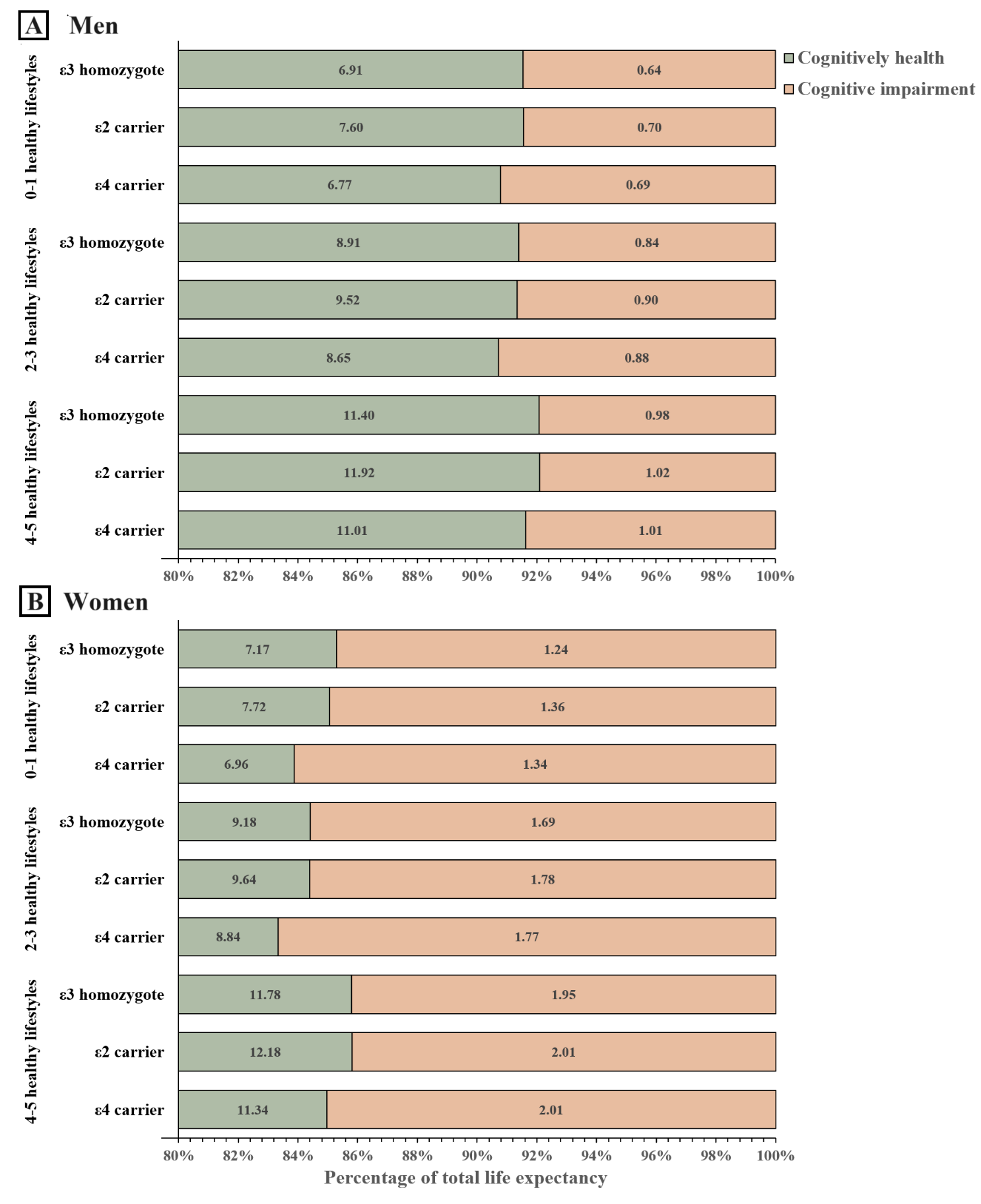


**Figure A.6. Life expectancies and proportions of cognitively health and cognitive impairment state at age of 85 by different APOE genotype and healthy lifestyles among both sexes. A: Men; B: Women.**


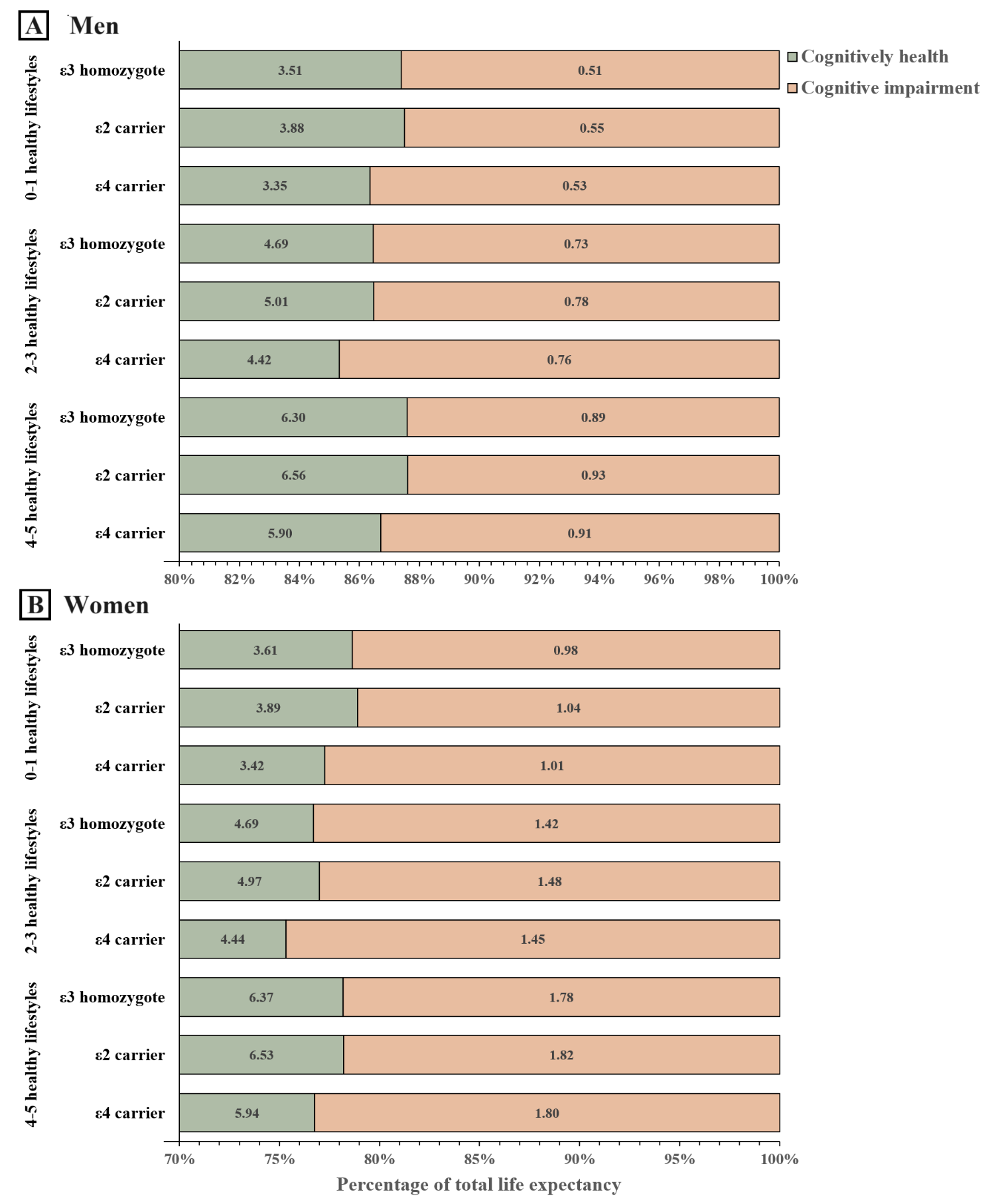

Supplement: Supplementary file 1 — Supporting Information [file ALZ-21-e70090-s002.docx]
